# Genetic and clinical insights into *MAST4*-related neurodevelopmental disorders

**DOI:** 10.3389/fped.2025.1603050

**Published:** 2025-06-27

**Authors:** Xiaohong Zheng, Foyang Fan, Bin Lei, Yao Xu, Min Peng, Youfeng Zhou

**Affiliations:** ^1^ College of Clinical Medicine for Obstetrics & Gynecology and Pediatrics, Fujian Medical University, Fujian Children’s Hospital (Fujian Branch of Shanghai Children’s Medical Center), Fuzhou City, China; ^2^ Research Department, Chigene (Beijing) Translational Medical Research Center Co., Ltd., Beijing, China

**Keywords:** MAST4, developmental delay, myelination dysplasia, *de novo* variant, whole-exome sequencing

## Abstract

**Objective:**

*De novo* variants in *MAST4* are increasingly implicated in neurodevelopmental disorders (NDDs), but the associated phenotypic spectrum remains incompletely characterized. We report a Chinese child with global developmental delay (GDD) and a novel MAST4 variant, further delineating the genotype-phenotype correlations for this gene.

**Methods:**

Clinical and genetic data were retrospectively analyzed for a proband diagnosed with a *MAST4*-related NDD at Fujian Children's Hospital. Trio-based whole-exome sequencing (WES) and subsequent Sanger sequencing were performed to identify and validate the pathogenic variant.

**Results:**

The 4-year-old male proband exhibited GDD with intellectual, motor, and speech impairments. Brain MRI showed delayed myelination. WES revealed a heterozygous *MAST4* missense variant (NM_001164664.2: c.4142G>T, p.Arg1381Leu), absent in population databases (gnomAD) and confirmed as *de novo*. The variant affects a highly conserved residue, supporting its likely pathogenicity. Phenotypic comparison with five previously reported cases confirmed core features of GDD and white matter abnormalities, though our patient lacked infantile spasms, underscoring clinical heterogeneity.

**Conclusion:**

This study reinforces *MAST4*'s role in NDDs and expands the genetic and phenotypic spectrum associated with this gene. The absence of infantile spasms in our case suggests variable expressivity, necessitating further functional studies to assess the variant's pathogenicity and *MAST4*'s neurobiological mechanisms.

## 1 Introduction

Neurodevelopmental disorders (NDDs) represent a heterogeneous group of conditions characterized by impairments in cognition, communication, behavior, and motor function, typically manifesting in early childhood. With a global prevalence of 8%–20%, NDDs impose substantial lifelong burdens on affected individuals and healthcare systems (1–4). Current diagnostic challenges stem from phenotypic heterogeneity and unreliable biomarkers, often delaying intervention. Moreover, treatment strategies remain largely symptomatic rather than mechanistic, underscoring the urgent need to elucidate the molecular underpinnings of these disorders. Advances in genetic research have revealed the remarkable complexity of NDD pathogenesis, involving diverse risk genes (e.g., *FMR1*, *SHANK3*) and variant types (e.g., copy number variations, single-nucleotide variants) (5–7). Notably, variable expressivity and incomplete penetrance complicate genotype-phenotype correlations, suggesting that additional modifiers may contribute to disease mechanisms.

Among emerging NDD-associated genes, the MAST family (*MAST*1–4) encodes microtubule-associated serine/threonine kinases with critical roles in neural development. These proteins share 49%–64% sequence homology and contain four conserved domains: DUF1908, a serine/threonine kinase domain, an AGC kinase C-terminal domain, and a PDZ domain (8). *MAST1* variants are linked to megacorpus callosum syndrome and ID (9), while *MAST3* variants are associated with NDDs with or without seizures (10). In contrast, *MAST2* remains poorly characterized. *MAST4* has garnered particular interest due to its multifaceted roles in neurodevelopment. Firstly, *MAST4* modulates microtubule dynamics and axonal integrity by activating Cdc42 via interaction with Tctex-1, a process critical for primary cilium resorption and synaptic signaling (11). Furthermore, through phosphorylation of FOXO1, *MAST4* suppresses *RTKN2* promoter binding, potentially influencing neuronal survival and synaptic plasticity (12). *MAST4* is highly expressed in oligodendrocytes, suggesting a role in myelination, a hypothesis supported by frequent white matter abnormalities in patients with *MAST4* variants (12, 13). Spatiotemporal expression analyses reveal that *MAST4* is enriched in the medial prefrontal cortex and thalamic nuclei during embryogenesis, with persistent expression in hippocampal neurons, cerebellar Purkinje cells, and oligodendrocytes in adulthood (8, 13). This pattern aligns with its putative functions in circuit formation and maintenance. Clinical evidence further implicates *MAST4* in NDDs, epilepsy, and hippocampal malformations (8, 14), though the phenotypic spectrum remains incompletely defined.

Here, we report a pediatric case featuring global developmental delay (GDD) and a novel *MAST4* variant. We combine clinical phenotyping with neuroimaging and genetic analyses to expand the genotype-phenotype landscape of *MAST4*-related disorders.

## 2 Materials and methods

### 2.1 Study participant and clinical evaluation

The proband was a 4-year-old male who presented to the Neurology Clinic of Fujian Provincial Children's Hospital at 2 years and 1 month of age with global developmental delay (GDD), including deficits in fine/gross motor skills and speech. Clinical assessment included neurological examination, developmental evaluation with China Developmental Scale for Children (CDSC) (0–6 years), and neuroimaging with cranial MRI. Electroencephalography (EEG) was deferred due to no clinical seizure history. The patient received intermittent neurodevelopmental therapy (focused on motor coordination and speech habilitation) through the hospital's rehabilitation program.

### 2.2 Genetic testing and variant interpretation

Trio whole-exome sequencing was performed by Chigene Translational Medicine Research Center Co., Ltd. (Beijing, China) on peripheral blood-derived DNA (proband and parents) using the IDT xGen Exome Research Panel v2.0 on an Illumina NovaSeq 6,000 platform (150 bp paired-end reads, average 100 × coverage). Raw reads were processed (fastq), aligned to GRCh37/hg19 (BWA-MEM v0.7.17), and variants called following GATK (v4.2.6.1) best practices, including duplicate marking, base quality recalibration, and local realignment. Variants were annotated (ANNOVAR) and filtered against population databases (gnomAD/ExAC/1,000 Genomes), then prioritized based on predicted impact using multiple algorithms (SIFT/PolyPhen-2/CADD/REVEL for missense; SpliceAI/dbscSNV for splicing; GERP++/phyloP for conservation). Variants were classified per ACMG/AMP guidelines with ClinGen specifications, integrating population frequency, computational predictions, inheritance patterns, and phenotype correlation (HGMD/ClinVar/OMIM). The *MAST4* variant (c.4142G>T) was validated by Sanger sequencing (ABI 3730xl).

## 3 Results

### 3.1 Clinical description

The patient, a 4-year-old boy, presented to our institution at 2 years and 1 month of age with global developmental delay affecting fine motor, gross motor, and speech domains. His clinical features included independent ambulation with gait instability, impaired manual dexterity (manifested by slow reaching/grasping and weak grip strength), and significant language delay (limited to single-word utterances with poor articulation). On physical examination, he was alert and responsive with stable vital signs. His head circumference measured 50.5 cm (within normal range), and his anterior fontanelle was closed. Skin examination revealed no jaundice, pallor, edema, or petechiae, with the absence of transverse palmar creases. Facial features included normal ocular motility without hypertelorism or ptosis, along with a mildly flattened nasal bridge. Oropharyngeal structures were intact, without a high-arched palate or cyanosis. Cardiopulmonary and abdominal assessments were unremarkable. Neurological evaluation demonstrated preserved visual and auditory responses, absent startle reflex, mild hypotonia, and symmetrically normal deep tendon reflexes (knee and Achilles ++). Plantar responses (Babinski sign), ankle clonus, and meningeal signs (Kernig's and Brudzinski's signs) were all negative.

**Figure 1:**
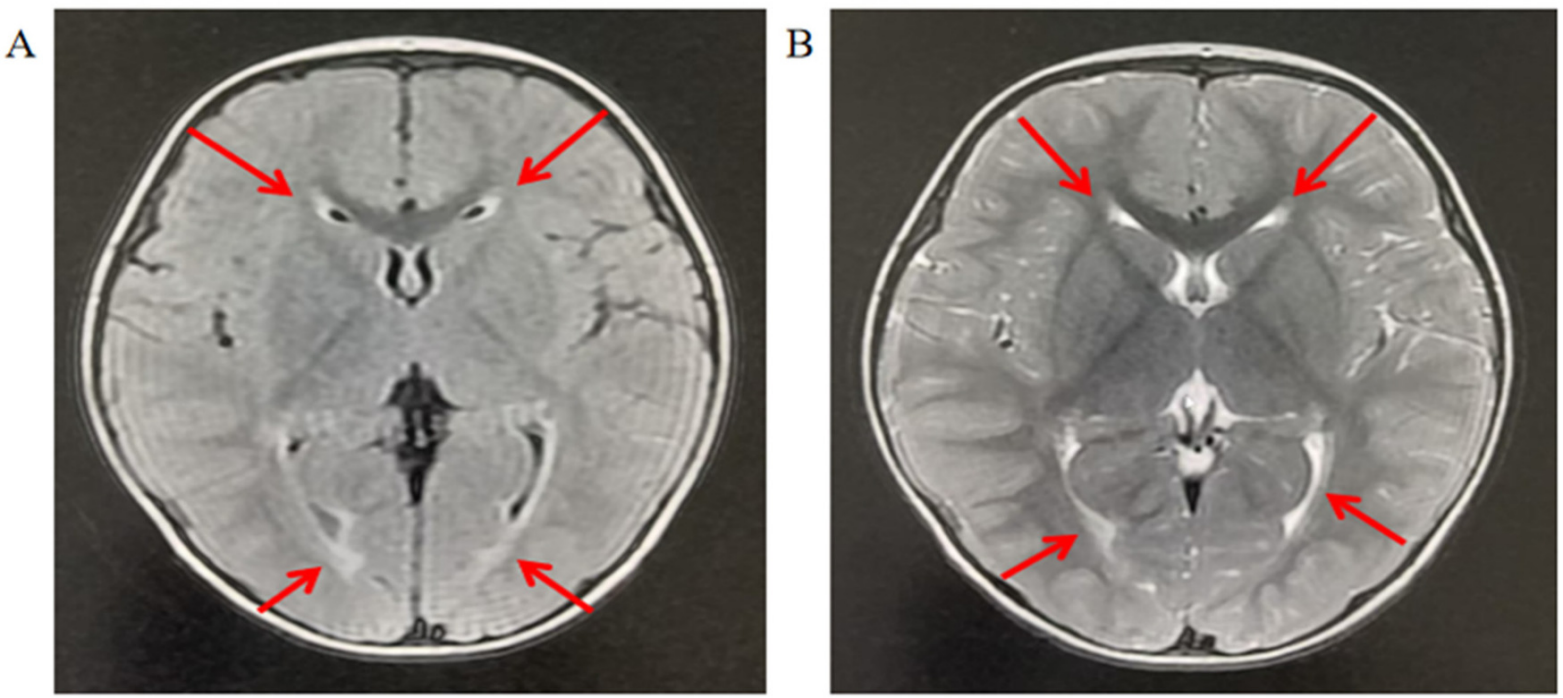
Brain MRI of the patient indicates myelination dysplasia. **(A)** T2-weighted imaging: The arrowhead denotes a region displaying abnormally persistent hyperintensity, suggesting delayed or disrupted myelination. This finding is consistent with hypomyelination, characterized by reduced myelin content and disorganized axonal maturation. **(B)** T1-weighted imaging: The arrow indicates a region with persistently hyperintense signal, further corroborating hypomyelination as immature white matter retains high T1 signal intensity until full myelination is achieved. The T1–T2 signal discrepancy (“T1 bright, T2 bright” paradox) is pathognomonic for developmental white matter abnormalities.

The child was born at term with a normal birth weight of 3.1 kg. During the neonatal period, he exhibited heightened startle reflexes and frequent, unexplained crying spells. His motor developmental milestones were as follows: head lifting in the prone position by 2–3 months, stable head control by 4 months, independent sitting by 10 months, crawling by 17 months, and unaided walking by 19 months. At 2 years and 1 month of age, he was referred to our outpatient clinic for evaluation of developmental delays in fine motor skills, gross motor function, and language abilities. A cranial MRI at that time revealed inadequate myelination (Figure 1).

Developmental assessment using the CDSC (0–6 years) for Children at 2 years 1 month and 3 years 6 months revealed persistent delays across all domains (Table 1). This assessment evaluates gross motor skills, fine motor skills, adaptive behavior, language competence, and personal-social behavior. The developmental quotient (DQ) serves as an indicator of overall developmental status, with classifications as follows: DQ ≥ 85 (normal development), 76–84 (borderline delay), 55–75 (mild delay), 40–54 (moderate delay), 25–39 (severe delay), and ≤24 (profound delay). In this case, the patient demonstrated persistent developmental delays, with both DQ scores falling below 70, consistent with mild developmental delay. This sustained subthreshold performance underscores the need for targeted clinical interventions to address deficits across multiple functional domains.

**Table 1:** China developmental scale for children (CDSC) (0–6 years).

Check the date and the project	Gross motor	Fine motor skills	Adaptive ability	Language ability	Social behavior	Total developmental quotient
2 years and 1 month of age	81.5	39.8	85.5	56.4	52.3	63.1
3 years and 6 months of age	7.46	38.1	84.4	57.15	53.34	62.49

### 3.2 Molecular data

**Figure 2:**
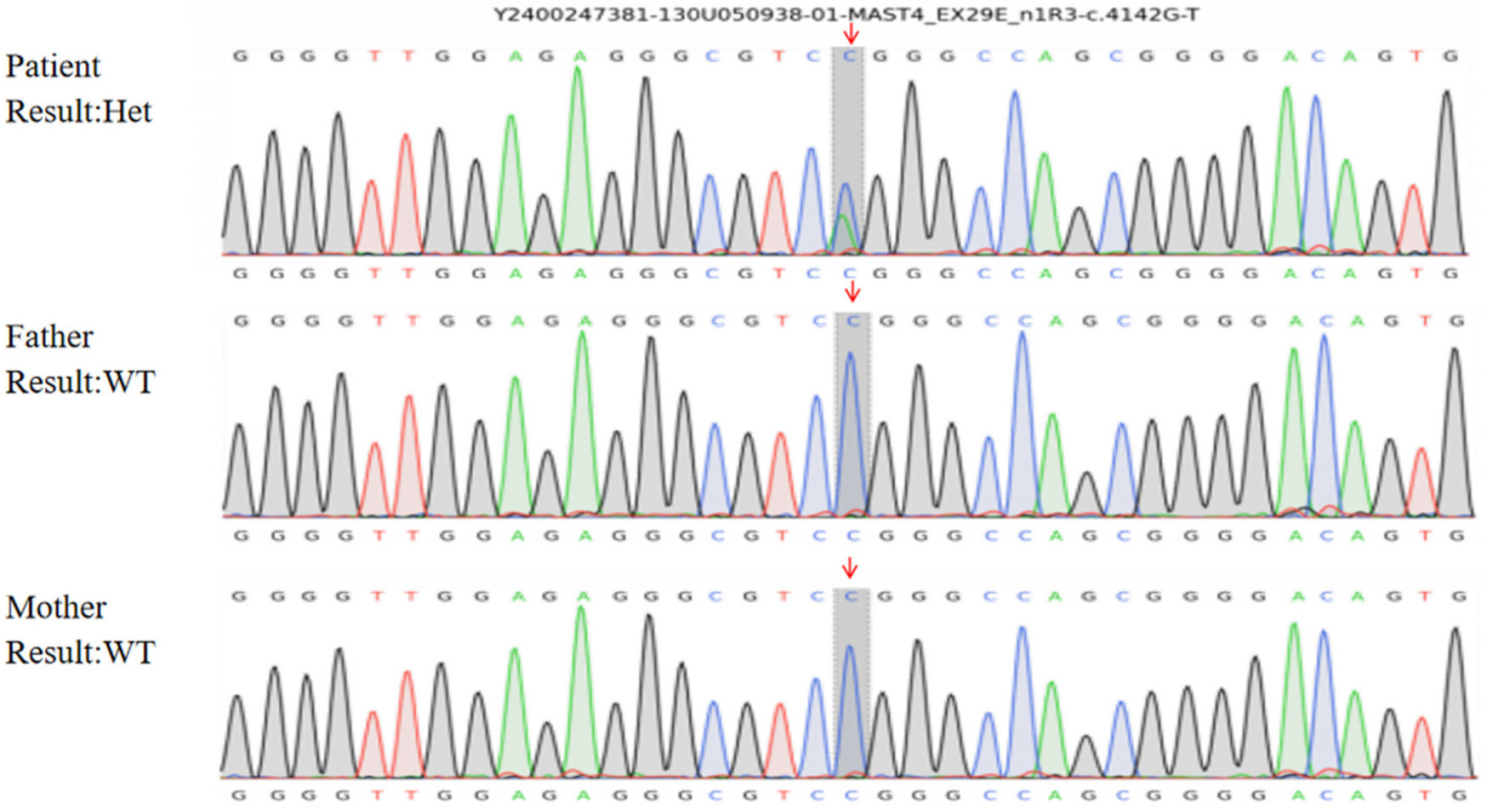
Sanger sequencing confirmation of the *MAST4* variant. Chromatogram showing the heterozygous c.4142G>T (p.Arg1381Leu) variant in the proband (indicated by overlapping G/T peaks at position 4,142). Both parents show wild-type sequences (G/G), confirming the *de novo* origin of this variant.

**Figure 3:**
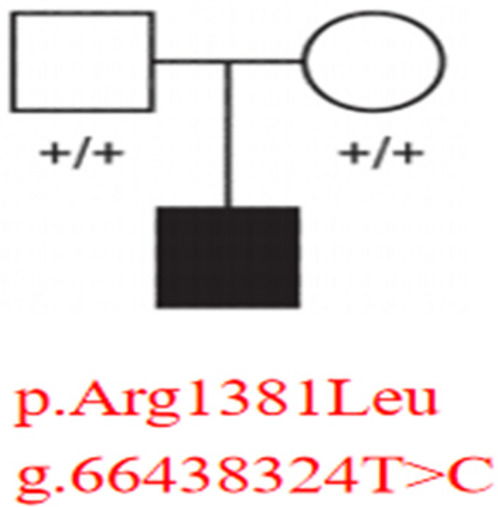
Pedigree analysis of the *MAST4* variant inheritance. Squares (male) and circles (female) represent family members, with filled symbols indicating affected status. The proband carries the heterozygous *MAST4* p.Arg1381Leu variant (g.66438324T>C), while both unaffected parents show wild-type genotypes (“+/+”).

Whole-exome sequencing identified a heterozygous *MAST4* missense variant (NM_001164664.2: c.4142G>T, p.Arg1381Leu) in the proband. Trio analysis confirmed its *de novo* origin (PS2), with Sanger sequencing demonstrating the variant in the proband (heterozygous G/T peaks) and wild-type genotypes (G/G) in both parents (Figure 2). Pedigree analysis revealed an autosomal dominant inheritance pattern with no family history of neurodevelopmental disorders (Figure 3). The variant was absent in major population databases [gnomAD v4.0 (807,162 alleles; 62,784 East Asian), ExAC, and ESP6500], with an allele frequency <0.0001% (PM2_Supporting). Computational tools unanimously predicted pathogenicity: SIFT (deleterious, 0.02), PolyPhen-2 (probably damaging, 0.998), REVEL (pathogenic, 0.88), and CADD (Phred 28.5) (PP3). The variant meets ACMG-AMP criteria for Likely Pathogenic classification (PS2 + PM2_Supporting + PP3). No other pathogenic variants or CNVs were detected that could explain the phenotype.

**Figure 4:**
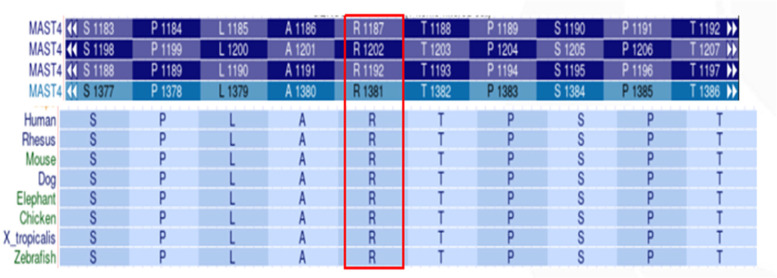
Evolutionary conservation analysis of *MAST4* p.Arg1381. Multiple sequence alignment shows high arginine residue conservation at position 1,381 across vertebrate species.

**Figure 5:**
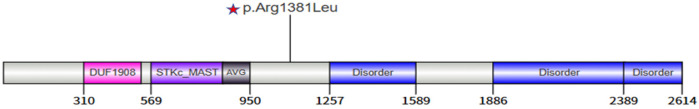
Domain architecture of *MAST4* protein showing the location of the p.Arg1381Leu variant.

Multiple sequence alignment revealed exceptional evolutionary conservation of Arg1381 across vertebrate species, suggesting critical functional importance (Figure 4). Structural modeling localized the variant to a disordered loop region (residue 1,381) (Figure 5).

**Figure 6:**
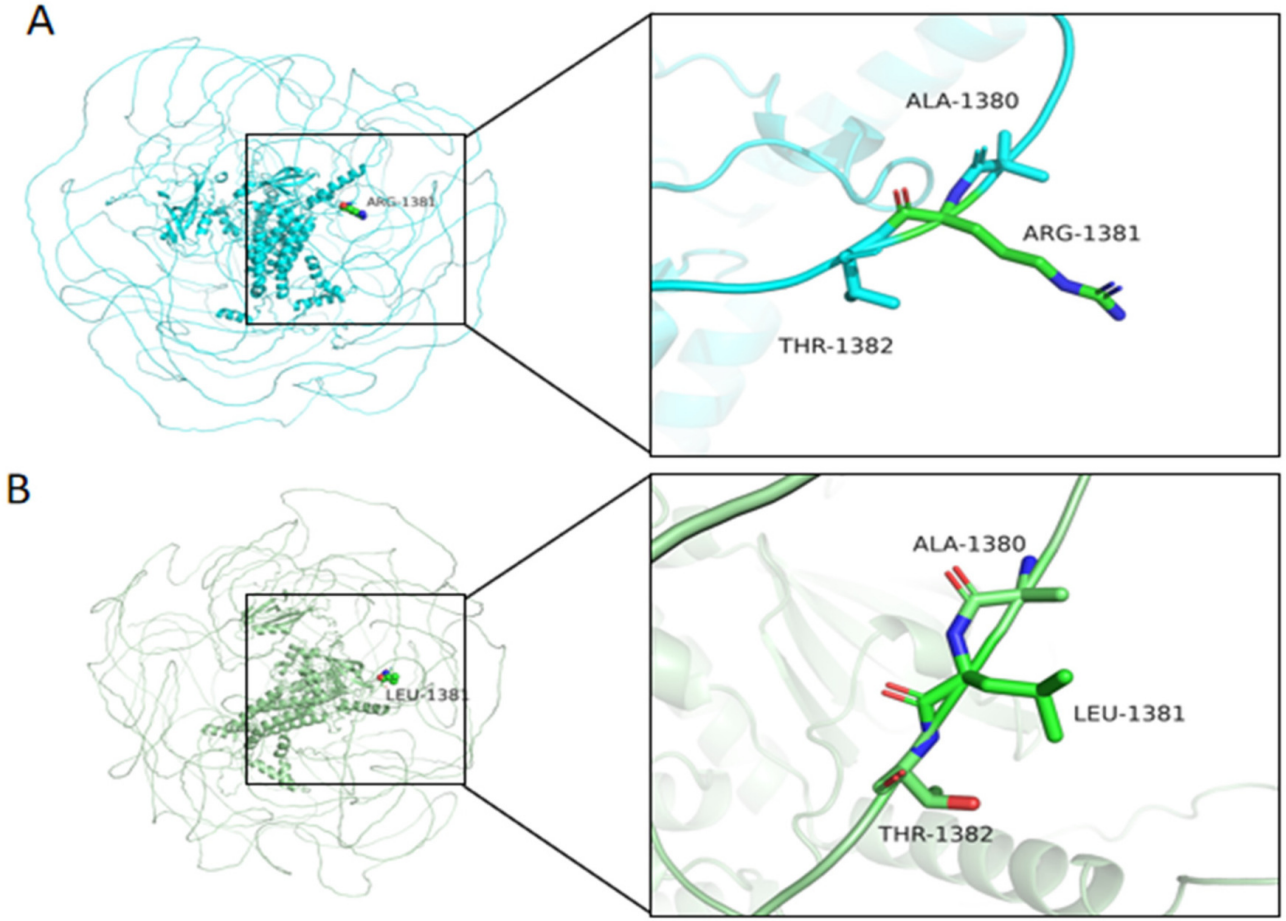
Structural comparison of wild-type and mutant *MAST4* protein at residue 1,381. **(A)** Wild-type MAST4 structure showing Arg1381 forming hydrogen bonds (dashed lines) with adjacent residues Ala1380 and Thr1382. **(B)** Mutant MAST4 (p.Arg1381Leu) structure demonstrating altered local conformation, potentially affecting protein stability or partner binding. Residue labels indicate positions 1,380–1,382 in both structures. Cyan, WT, green, mutant.

AlphaFold2-based modeling (Google DeepMind) revealed that wild-type *MAST4* Arg1381 forms stabilizing hydrogen bonds with Ala1380 and Thr1382 in the kinase domain (Figure 6A). While the p.Arg1381Leu variant maintained similar hydrogen bond numbers, the substitution of a positively charged arginine with hydrophobic leucine likely impairs function through (Figure 6B): (1) disruption of local electrostatic balance, potentially affecting substrate binding; and (2) steric interference with kinase domain accessibility. This charge and structural alteration at a highly conserved residue may compromise protein-protein interactions and catalytic activity, suggesting a plausible mechanism by which this variant contributes to neurodevelopmental pathology through structure-function perturbation. Together, these findings provide strong evidence for the pathogenic potential of this *de novo MAST4* variant in the patient's neurodevelopmental disorder.

### 3.3 Literature review

#### 3.3.1 *MAST4* in neurodevelopmental disorders and epilepsy

Through a systematic review of PubMed literature using the search term “*MAST4* gene variant”, we identified seven relevant publications, including two studies definitively linking *MAST4* variants to neurodevelopmental disorders. Table 2 summarizes the clinical characteristics of patients with *MAST4* variants from previous reports and the current case. Cases 1–4 were derived from the prior study (8), while Case 5 represents the novel clinical presentation reported in this investigation. The seminal study (8) identified four children harboring *de novo MAST4* missense variants (p.Ile898Thr, p.Thr1471Ile, and p.Ser2552Trp), all exhibiting global developmental delay and intellectual disability, with three patients demonstrating infantile spasms—two with drug-resistant epilepsy and one achieving seizure remission on levetiracetam. Structural brain anomalies, including ventriculomegaly and corpus callosum hypoplasia, were observed in a subset of cases. These variants, absent from all major population databases (gnomAD/ExAC/ESP6500), were unanimously predicted as pathogenic by multiple computational algorithms (SIFT/PolyPhen-2/REVEL/CADD). The clinical presentations consistently featured neurodevelopmental impairments coupled with diverse seizure phenotypes, including drug-responsive focal seizures and developmental epileptic encephalopathy. Landoulsi et al. (15) support *MAST4*'s role in epileptogenesis through its critical functions in microtubule-associated kinase activity and neuronal signaling pathways. The collective evidence positions *MAST4* as a clinically relevant gene in the overlapping spectra of neurodevelopmental disorders and epilepsy, with particular importance for cases presenting with infantile spasms and developmental delay.

**Table 2:** The clinical features of the affected individual with the *MAST4* variants.

Individuals	Case1	Case2	Case3	Case4	Case5
Variant	c.2693T>C (p.Ile898Thr)	c.4412C>T (p.Thr1471Ile)	c.4412C>T (p.Thr1471Ile)	c.7655C>G (p.Ser2552Trp)	c.4142G>T (p.Arg1381Leu)
Inheritance	*De novo*	*De novo*	*De novo*	*De novo*	*De novo*
Sex	Female	Male	Male	Female	Male
Age at exam	1 y 7 m	2 y 5 m	2 y 3 m	3 y 3 m	2 y 1 m
Motor delay	−	+, Moderately	+, Moderately	−	+, Mildly
Speech delay	+, Mildly	+, Moderately	+, Moderately	+, Mildly	+, Mildly
Intellectual disability	+, Mildly	+, Moderately	+, Mildly	+, Moderately	+, Mildly
Seizures	Infantile spasms	Infantile spasms	Infantile spasms	Infantile spasms	Seizure-free
Epilepsy controlled	poorly controlled	well-controlled	well-controlled	poorly controlled	Seizure-free
EEG	Multiple spike waves mixed with irregular slow waves and low amplitude fast waves with voltage decay	Widespread spike slow waves with low amplitude and the voltage decay	Generalized spike-slow waves following diffuse voltage decay	Poly spike-slow and irregular slow waves, partial hypsarrhythmia	EEG was not performed
Brain MRI	Widened extra cerebral interspace of bilateral frontal and temporal lobes, dilatation of the bilateral lateral ventricles, and dysgenesis of the corpus callosum	Bilateral ventriculomegaly, Widened bilateral frontotemporal; Sulci and fissures; bilateral hippocampal MRS asymmetry, right NAA	Reduced white matter, dilatation of the bilateral lateral ventricles, delayed myelination of the posterior limb of the internal capsule, and occipital lobe white matter.	Sharp frontal anterior skull, decreased anteroposterior diameter of the skull.	inadequate myelination
Sleep disturbance	NA	+, Mildly	+, Mildly	NA	−
Facial dysmorphism	Normal	A wide nasi, gothic arch, penetrating palm	Penetrating palm, bulbous nose, low-set ears	Normal	Normal

Symbols: “+”, present; “−”, absent; NA, not available.

NAA, N-acetylaspartate, a neuroimaging marker of brain metabolic activity.

Widened extra-cerebral interspace: Enlargement of CSF spaces between brain and skull, potentially indicating neurodevelopmental delay.

#### 3.3.2 *MAST4* involvement in non-neurological conditions

The remaining five articles revealed *MAST4*'s pleiotropic roles beyond the nervous system. In developmental disorders, *MAST4* variants were found to inhibit the Wnt/β-catenin signaling pathway, leading to enamel hypoplasia through functional interactions with known tooth agenesis-associated genes (e.g., *WNT10A*, *AXIN2*) (16). In oncology, variants in *MAST4* have been implicated in the progression of acral melanoma, potentially facilitating tumor cell metastasis through alterations in adhesion, motility, and invasiveness (17). Additionally, *MAST4* expression levels were integrated into kinase profiling for myelodysplastic syndromes (MDS), demonstrating prognostic value for disease progression risk stratification and therapeutic target responsiveness (18). In prostate cancer research, *MAST4* has been identified as a participant in chromosomal translocations within the 5q region of the VCaP cell line, a process driven by chromothripsis. This highlights *MAST4*'s potential role in contributing to genomic instability, as chromosomal translocations are a form of chromosomal instability that can lead to cancer development (19).

To summarize, *MAST4* variants exhibit remarkable pleiotropy, contributing to neurodevelopmental disorders with epilepsy (8, 15), enamel defects (16), cancer metastasis (17), MDS risk stratification (18), and chromosomal rearrangements in malignancies (19). However, our patient lacked infantile spasms and displayed delayed myelination as the primary neuroimaging feature, contrasting with the previously reported structural abnormalities. This phenotypic divergence suggests either variable expressivity of *MAST4* variants or modulation by genetic modifiers. This multifactorial involvement underscores the need for mechanistic studies to delineate tissue-specific pathogenic pathways and explore therapeutic targeting strategies.

## 4 Discussion

### 4.1 *MAST4* expression and function

*MAST4*, a member of the MAST kinase family, is crucial for neurodevelopment as it regulates axonal guidance and synaptic plasticity via its kinase domain. This regulation is part of the complex molecular mechanisms that ensure proper neural network formation and function. Its predominant expression in cerebellar Purkinje cells, hippocampal regions, and white matter-enriched areas (20), along with embryonic enrichment in the dorsal midline thalamic nuclei and medial prefrontal cortex (8, 21), underscores its importance in early neural circuit formation. Animal studies reveal seizure-induced upregulation of *MAST4* in the murine hippocampus (22), suggesting involvement in epileptogenic neuroplasticity. Functionally, *MAST4* modulates neuronal survival via FOXO1 phosphorylation and regulates ciliary dynamics through Tctex-1/Cdc42 interactions (8, 10), establishing it as a multifunctional orchestrator of neural development and homeostasis. All of these suggest that *MAST4* variants disrupt cellular self-renewal, proliferation, and differentiation (12).

### 4.2 Variant novelty and conservation

The pathogenicity of *MAST4* variants in NDDs remains poorly characterized, as no *MAST4*-related disorders are currently cataloged in OMIM. Although the Human Gene Mutation Database (HGMD) lists 23 *MAST4* variants classified as disease-causing (DM), their clinical significance lacks validation in large cohorts or functional studies. The *de novo MAST4* missense variant (c.4142G>T, p.Arg1381Leu) identified in our patient offers several lines of evidence supporting its potential pathogenicity: (1) The variant is absent in gnomAD v4.0, ExAC, and ESP6500, suggesting strong negative selection; (2) Arg1381 is highly conserved across vertebrates (Figure 4), implying functional indispensability in the *MAST4* kinase domain; (3) Concordant deleterious predictions from SIFT (deleterious), PolyPhen-2 (probabaly damaging), REVEL (0.885), and CADD (28.5) further bolster its disease association. Structural modeling localizes p.Arg1381Leu to a critical loop region within the kinase domain (Figure 5). The substitution of a charged arginine with a hydrophobic leucine likely destabilizes local protein conformation, impairing *MAST4*'s interaction with downstream substrates (Figure 6). Unlike *MAST1* variants, which are associated with megalencephaly and corpus callosum anomalies, our patient's *MAST4* variant correlates with delayed myelination and developmental delay, without structural malformations. This divergence may reflect differential substrate specificity among MAST kinases, with *MAST4* preferentially influencing white matter integrity via distinct signaling cascades.

### 4.3 Case interpretation and phenotypic comparison

Our patient presents a distinct clinical manifestation compared to previously reported *MAST4* cases, characterized by a unique combination of significant global developmental delay (DQ < 70) without epileptic manifestations and predominant delayed myelination on MRI (Figure 1), contrasting with the typical structural abnormalities seen in other cases. This phenotypic divergence likely results from the interplay between variant-specific effects and epigenetic modulation. The p.Arg1381Leu variant may disrupt distinct protein interaction networks and substrate recognition, creating a unique perturbation pattern. Importantly, epigenetic mechanisms appear to play a protective role against seizures while potentially exacerbating myelination defects. The adenosine-mediated DNA hypomethylation pathway (23) and histone modification cascades may maintain an anti-epileptic state, while disproportionate disruption of Wnt/β-catenin signaling (24) and mTOR-mediated OPC differentiation (25) likely contribute to the myelination delay. The involvement of SWI/SNF and CHD chromatin remodelers (26) in oligodendrocyte differentiation, along with the known neurodevelopmental roles of H3K4 trimethylation and H3K27 demethylation (27), suggests complex epigenetic modulation of myelination defects. Although direct evidence linking *MAST4* to these pathways requires further investigation, the kinase domain's potential to phosphorylate chromatin modifiers and its known regulation of cytoskeletal dynamics suggest plausible mechanistic connections. These findings highlight three key aspects of *MAST4* pathobiology: domain-specific effects, epigenetic buffering capacity, and developmental stage-dependent neural vulnerability, underscoring the need for integrated genetic-epigenetic analyses.

### 4.4 Implications for future research

Our findings highlight three critical avenues for further investigation: First, constructing *MAST4* p.Arg1381Leu mutant mouse models to systematically delineate its spatiotemporal effects on myelination and synaptic plasticity, with a focus on oligodendrocyte differentiation and axon-glia interactions. Second, integrating whole-genome sequencing and chromatin conformation capture (Hi-C) to identify *MAST4*-interacting epigenetic regulators (e.g., *HDAC4*, *MECP2*) and uncover molecular drivers of phenotypic heterogeneity. Third, developing targeted therapies, including allosteric small-molecule modulators or AAV-based gene editing strategies, to restore *MAST4* kinase functionality. These efforts will deepen mechanistic insights and accelerate translational applications for *MAST4*-related disorders.

## 5 Conclusion

*De novo* variants in *MAST4* are emerging as important genetic contributors to neurodevelopmental disorders, typically presenting with global developmental delay and white matter abnormalities. This case report broadens the known phenotypic spectrum by characterizing a novel p.Arg1381Leu variant and its unique association with myelination deficits in the absence of epilepsy—a departure from previously reported cases.The observed phenotypic heterogeneity likely reflects a complex interplay between the primary variant and modifier gene effects, particularly through epigenetic regulation of *MAST4* expression during neurodevelopment. For instance, variability in DNA methylation at SOX10-binding enhancers may influence oligodendrocyte progenitor cell (OPC) migration in a region-specific manner, while H3K27ac-mediated chromatin remodeling could mitigate the consequences of *MAST4* haploinsufficiency on axonal guidance pathways. This multilayered regulatory framework may explain both the developmental stage-specific expression of white matter abnormalities and the apparent resilience against epileptogenesis in this patient. Future directions should include: (1) Establishing multicenter cohorts to better delineate genotype-phenotype correlations. (2) Assessing functional impact through *in vitro* kinase activity assays and conditional knockout models. (3) Applying multi-omics approaches—such as single-cell methylome and 3D chromatin architecture profiling—to investigate epigenetic compensatory mechanisms in glial cells. (4) Developing brain organoid models using CRISPR-dCas9-based epigenetic editing to simulate modifier gene effects on neuron-glia interactions. (5) Conducting longitudinal follow-up to track neurodevelopmental trajectories and therapeutic responses.These efforts will refine molecular diagnostic strategies and pave the way toward personalized treatment approaches for *MAST4*-related neurodevelopmental disorders.

## Data Availability

The original contributions presented in the study are included in the article/Supplementary Material, further inquiries can be directed to the corresponding author.
